# Endoscopic Holmium Laser harvesting of bladder mucosal graft for substitution urethroplasty

**DOI:** 10.1590/S1677-5538.IBJU.2022.99.09

**Published:** 2021-12-14

**Authors:** Felipe C. A. Figueiredo, Luiz Augusto Westin Carvalho, Luís Otávio Pinto, Patrick Ely Teloken, Luciano Alves Favorito

**Affiliations:** 1 Universidade do Estado do Rio de Janeiro Departamento de Urologia Rio de Janeiro RJ Brasil Departamento de Urologia, Universidade do Estado do Rio de Janeiro - Uerj, Rio de Janeiro, RJ, Brasil; 2 Hospital dos Servidores do Estado do Pará Serviço de Urologia Reconstrutora Belém PA Brasil Serviço de Urologia Reconstrutora, Hospital dos Servidores do Estado do Pará, Belém, PA; 3 Sir Charles Gairdner Hospital Department of Urology Perth Australia Department of Urology, Sir Charles Gairdner Hospital, Perth, Australia; 4 Universidade do Estado do Rio de Janeiro Unidade de Pesquisa Urogenital Rio de Janeiro RJ Brasil Unidade de Pesquisa Urogenital - Universidade do Estado do Rio de Janeiro - Uerj, Rio de Janeiro, RJ, Brasil

## Abstract

**Introduction:**

Tissue transfer has been used in urethral reconstruction for decades, and several grafts have been described (1, 2). The ideal graft would have optimal tissue characteristics and lead to minimal morbidity at the donor site. Urethroplasty using bladder mucosa was first described by Memmelaar in 1947 (3). The main limitation in using bladder mucosal grafts has been the invasiveness of open harvesting (4). We describe an endoscopic technique using Holmium: YAG laser to harvest bladder mucosal graft for substitution urethroplasty.

**Methodology:**

A 33-year-old male with no history of urethral instrumentation, trauma, or infection presented with obstructive lower urinary tract symptoms. On retrograde urethrogram a 6cm bulbar urethral stricture was identified. Several options were discussed, and the patient opted for a one-sided onlay dorsal urethroplasty (5) using a bladder mucosal graft.

Equipment used to harvest the graft included an 18.5Fr continuous flow laser endoscope with a Kuntz working element (RZ) and a 60W Holmium Laser (Quanta) with 550μm laser fiber.

The procedure was started by making a perineal incision, urethral mobilization and incision of the stricture segment. The laser endoscope was then introduced via the perineum. Settings of 0.5J, 30 Hz, and long pulse were used and a 7 x 2.5cm graft was harvested from the posterior bladder wall. Hemostasis of the harvest site was performed. The bladder mucosal graft was thinned in similar fashion to a buccal mucosal graft and sutured as per previously described techniques.

**Conclusion:**

Endoscopic Holmium Laser harvesting of bladder mucosal graft is feasible and may allow this graft to become an alternative to buccal mucosa. Further studies are required to define its role in urethral reconstruction.

**Figure:** Available at:http://www.intbrazjurol.com.br/video-section/20229909_Figueiredo_et_al
